# The Embodiment of Objects: Review, Analysis, and Future Directions

**DOI:** 10.3389/fnins.2019.01332

**Published:** 2019-12-13

**Authors:** Aubrie Schettler, Vicente Raja, Michael L. Anderson

**Affiliations:** ^1^Department of Philosophy, Western University Canada, London, ON, Canada; ^2^Rotman Institute of Philosophy, Western University Canada, London, ON, Canada; ^3^Brain and Mind Institute, Western University Canada, London, ON, Canada

**Keywords:** embodiment, prosthethics, virtual reality, body schema, body image

## Abstract

Here we offer a thorough review of the empirical literature on the conditions under which an object, such as a tool or a prosthetic (whether real or virtual), can be experienced as being in some sense a part or extension of one’s body. We discuss this literature both from the standpoint of the apparent malleability of our body representations, and also from within the framework of radical embodied cognition, which understands the phenomenon to result not from an alteration to a representation, but rather from the achievement of a certain kind of sensory/motor coupling. We highlight both the tensions between these frameworks, and also areas where they can productively complement one another for future research.

## Introduction

Is it possible to “embody” external objects, such as prosthetics or tools, that is, to treat or regard them as in some important sense actually part of our bodies? Most of the literature concludes that people can extend the borders of the physical body to temporarily incorporate different prosthetics, such as rubber hands, into their body image (i.e., their conscious beliefs regarding their bodies; see Botvinick and Cohen, 1998; Ehrsson et al., 2004, 2005, 2008; Tsakiris and Haggard, 2005; Tsakiris et al., 2006; Marasco et al., 2011; D’Alonzo and Cipriani, 2012; D’Alonzo et al., 2014; Collins et al., 2016), and certain external objects, such as tools, into their body schema (i.e., their unconscious knowledge of their bodies and its capacities; see Cardinali et al., 2009b, 2012; Sposito et al., 2012; Baccarini et al., 2014; Garbarini et al., 2015). Several studies indicate that there are surprisingly few constraints on incorporating objects into either of these two kinds of body representations.^1^ In fact, along with using real life tools, even imagining using tools can cause them to be incorporated into the body schema (Baccarini et al., 2014).

In this review, we highlight neuropsychological research and the numerous illusion studies on neurotypical individuals that explore embodiment, including those that use the rubber hand paradigm and/or virtual reality setups. The rubber hand illusion has been a paradigm widely used in studies of the relationship between the body image and the body schema. In the rubber hand illusion (detailed further in the next sections) an experimenter simultaneously touches a suitably positioned fake hand and the participant’s real hand hidden from view. The effect is participants feel the rubber hand to be their own. Virtual reality environments have also been used to investigate embodiment phenomena, and offer flexibility to address how and to what extent objects and tools are experienced and incorporated into our body representations. It is flexible in the sense that different virtual environments can be created to test the incorporation of objects and tools into our body representations and virtual body parts and bodies can be manipulated differently than in other non-virtual reality experimental setups. It also allows for one’s “objective body to remain in the real world, while one’s phenomenal body can be projected into terminal reality” (Murray and Sixsmith, 1999). Namely, that while one is immersed in a virtual environment, at least several aspects of her phenomenal body (e.g., the visually perceived body) are part of the virtual environment: one visually perceives whatever body she has in the virtual environment and not her body in the real world.

In this paper, first we outline two competing frameworks for understanding object and tool embodiment and review the relevant literature of embodiment of objects and tools from the point of view of these two fundamental frameworks. In Sections “Embodying Objects” and “Embodying Tools,” respectively, we analyze the way object and tool embodiment are understood in terms of body representations. In both cases we analyze experimental paradigms such as the rubber hand illusion and the use of virtual environments. In Section “Radically Embodied Tools and Objects,” we analyze object and tool embodiment from the specific point of view of radical embodiment. In all cases we pay special attention to the deeper theoretical commitments and the experimental methodology of the different frameworks. Importantly, our aim is not to critically analyze one of these two frameworks from the point of view of the other one, but to review them and to explore their compatibilities and incompatibilities.

## Embodiment

Writ large, the embodiment of objects and tools may be defined as the sense that those objects and tools has become “part of us” in a similar way that our limbs or our fingers are parts of us. More specifically, the embodiment of objects and tools includes the sense of ownership, the sense of agency, and the sense of self-location (Kilteni et al., 2012), which often are characterized as *affective*, *motor*, and *spatial* embodiment, respectively (de Vignemont, 2011).^2^ We turn to them now to offer a brief introduction. The main concepts will be revisited and elaborated in later sections.

### Affective, Motor, and Spatial Embodiment

The sense of ownership or *affective embodiment* refers to a situation where an individual shows the same affective reactions for the object as for their own body (de Vignemont, 2011). In this sense, affective embodiment is one of the fundamental components of the embodiment of objects and tools: the first step toward such an embodiment is the actual sense that a given object or tool is part of one’s body. Measures of affective embodiment involve evaluating behavioral and physiological responses in situations in which an object may be said to be embodied.

The sense of agency or *motor embodiment* corresponds to the situation where an object or tool “is processed in the same way as a part of one’s body for motor tasks” (de Vignemont, 2011, p. 87). Measures of motor embodiment are more often used in virtual reality and tool embodiment studies. If “the motor system takes the properties of [a tool] into account as properties of the effector in planning, then [it] is embodied” (de Vignemont, 2011, p. 86). Underlying the sense of agency is a modulation of the body schema. For instance, after tool use there can be consequences on free hand movement kinematics (Cardinali et al., 2009b).

The sense of self-location or *spatial embodiment* involves a bodily frame, an external frame, and a peri-personal frame (de Vignemont, 2011). The bodily frame is the body space’s boundaries. For instance, if an object “is taken into account by the representation of the body space, by replacing a missing body part, by adding a new body part, or by stretching an existing body part, then [it] can be said to be embodied” (de Vignemont, 2011, p. 85). Moreover, localizing “bodily sensations in [an object] shows that [it] is taken into account by the representation of the body space and that [it] is embodied” (Ibid.). For instance, amputees can sometimes feel things in contact with their prosthetic (Murray, 2004). The external frame is represented the mis-localization of a bodily object toward a non-bodily object (aka *proprioceptive drift*). For instance, if “the location of [an object] within the external frame is processed in the same way as the location of a part of one’s body, then [it] is embodied” (de Vignemont, 2011, p. 85). Lastly, the peri-personal frame “is the frame of the space immediately surrounding a specific part of one’s body (<30–50 cm)” (de Vignemont, 2011, p. 85). An object or tool is embodied if the object or tool is processed as peri-personal space (de Vignemont, 2011).

It is worth noting that the three senses of embodiment are not independent from each other. For example, brain studies and movement disorders seem to show that there is a mutual relationship between affective embodiment and the motor system (Schütz-Bosbach et al., 2006; Della Gatta et al., 2016; Burin et al., 2017; Fossataro et al., 2018). Affective embodiment requires multisensory integration within a fronto-parietal network. This network includes an important area for the interaction between affective embodiment and motor system, the ventral premotor cortex (Ehrsson et al., 2004; Makin et al., 2008b; Blanke et al., 2015). Also, spinal cord injuries have been shown to have an altered sense of body ownership (Scandola et al., 2014).

Additionally, affective embodiment and some aspects of spatial embodiment, such as proprioceptive drift, reflect different components of the multisensory integration process (Ehrsson et al., 2005; Longo et al., 2008; Makin et al., 2008a; Rohde et al., 2011; Martinaud et al., 2017). For example, fMRI data shows there is a relationship between ventral premotor activation and subjective report, which is related to affective embodiment (Ehrsson et al., 2004) and inferior parietal lobule activation during recalibration of perceived hand position, which is related to proprioceptive drift (Ehrsson et al., 2004, 2005).

Finally, it is important to note the influence of perspective for embodiment although it is still a matter of discussion. On the one hand, some studies have suggested that the first-person perspective is important for embodiment (Blanke and Metzinger, 2009), which seems to be confirmed in rubber hand experiments (Pavani et al., 2000; Kalckert and Ehrsson, 2012; Bucchioni et al., 2016) and full body virtual reality experiments (Petkova and Ehrsson, 2008; Slater et al., 2010; Petkova et al., 2011; Maselli and Slater, 2013). Also, researchers found with the use of a questionnaire (Tieri et al., 2015b), measuring skin conductance response (Tieri et al., 2015a), and electroencephalographic response (Pavone et al., 2016), that passively observing a virtual body in first-person perspective is sufficient for embodiment (Maselli and Slater, 2013; Tieri et al., 2015b, 2017). A similar result found that “visual capture by a fake hand (without any synchronous or asynchronous tactile stimulation) affects body ownership in a group of hemiplegic patients with or without disturbed sensation of limb ownership” (Martinaud et al., 2017, p. 174). On the other hand, others do not observe significant differences between first and third person perspectives for embodiment (Pomés and Slater, 2013).

### The Cognitive Science of Embodiment: Two Approaches

When it comes to understanding how we embody objects or tools, two wide theoretical frameworks are used in contemporary research. One framework regards the embodiment of objects or tools as a matter of incorporating them into our body representations, that is the body schema, and/or image. The other framework, that will be referred as radical embodiment, takes the process of embodiment to be the constitution of a new complex system with both somatic and extrasomatic components.

The framework based on body representations posits the existence of some kind of body model or models that represent diverse features of the body and the way non-bodily objects and tools may be integrated into those representations. A way to categorize these representations is to refer to them as the *body image* or one’s perceptions, beliefs, and attitudes toward their body, and the *body schema* or one’s motor capacities that work without conscious appraisal (Gallagher and Meltzoff, 1996). The body image is often characterized as a cognitive appraisal, perception, or evaluation of one’s own appearance and body shape, and the related or resulting affect (Levine and Smolak, 2014). The body schema, in contrast, is a non-conscious neural map of the spatial relations among the body parts and the body’s motor capacities. The body schema is a plastic representation (Giummarra et al., 2008; Gallagher, 2013) and is constantly updated due to incoming sensory input (Dijkerman and de Haan, 2007; Giummarra et al., 2008).

Although the dichotomy between body image and body schema is quite standard in the literature, it is by no means uncontested. Specifically, the relationship between body image and body schema as body representations is intensely debated. Some authors defend the existence of a unitary body representation that must encompass the features usually attributed to body images and body schemas as distinct representational entities (de Vignemont and Farnè, 2010). Support for this interpretation comes from the self not being experienced as many separate body parts, but as a single whole-body representation. This is important since the brain might resolve sensory conflicts by checking the compatibility of multisensory input with a prior body representation, which determines what can and cannot be embodied (Tsakiris and Haggard, 2005; De Preester and Tsakiris, 2009; Tsakiris, 2010; Moseley and Flor, 2012). This long-term body representation is an anatomical structure of the body and is continuously updated from the sensory modalities (Botvinick and Cohen, 1998; Naito et al., 2002; Lackner and Dizio, 2005). On this view, the process of “embodiment must respect some basic anatomical constraints. Therefore, only some objects [and tools] under certain circumstances can be processed as if they were parts of one’s body” (de Vignemont and Farnè, 2010, p. 205).

Alternatives to this unitary approach include the dyadic and triadic body representation models (Schwoebel and Coslett, 2005; Gallagher, 2013) or somatosensory streams (Dijkerman and de Haan, 2007). On the one hand, the dyadic model takes the body schema to be an action-based sensorimotor representation, and the body image to be a non-action-based body representation (Gallagher and Cole, 1995; Rossetti et al., 1995; Dijkerman and de Haan, 2007). This is the standard view presented before in this section. On the other hand, the triadic body representation model accepts the standard notion of body schema but dichotomizes the body image into two different representations: the body semantics and the body structural description representations (Schwoebel and Coslett, 2005). Another model has also further divided the body schema into a motor schema and a somatosensory schema so that there are at least five body representations (Anderson, 2018). Here, we will simply accept the simple dyadic model as sufficient for our purposes in framing the following discussion. As prosthetics become more sophisticated in their capacity to offer various kinds of somatic feedback, our understanding of how such objects can be embodied will have to be likewise updated.

The framework of radical embodiment does not appeal to the concept of body representations to understand the way objects and tools can be embodied. In general, radical embodiment takes cognitive systems to be a kind of complex, self-organized system in which many components interact with each other to give rise to a given cognitive ability (Van Orden et al., 2003; Chemero, 2009; Cavagna et al., 2010; Anderson et al., 2012). The different components of these cognitive systems extend through the brain, the body, and the environment; and, in this sense, object and tools of very different kinds may be parts of cognitive systems and, therefore, may be embodied in those systems just insofar as they are properly coupled to them.

Understanding object and tool embodiment in terms of the activity of a cognitive system extending beyond the brain and the body and integrating those objects and tools into that activity, is a common feature within some radical proposals in the embodied approach to cognition. The strongest forms of the hypothesis of the extended mind (Menary, 2010; Kirchhoff and Kiverstein, 2019) and those approaches based on ecological psychology, enactivism, and dynamic systems share this form of radical embodiment. In both cases, the signatures of the embodiment of objects and tools are not taken to be a matter of body representations, but a matter of coupling between the different components of the system (e.g., the body + prothesis, the body + a tool, and so on; see, e.g., Van Orden et al., 2005; Dotov et al., 2010). The nature and degree of such a coupling requires its own theoretical and methodological commitments to be fully understood.

## Embodying Objects

Physical objects are those things that are animate or inanimate and can persist through time, such as a car, a pen, or a prosthetic device. Objects are affected by external forces, can be threatening or non-threatening, and can be cognitively reflected on Longo (2016). Some views on the body take it to be a multisensory object that seems to obey our will, has the ability to interact with other objects, can incorporate them, and can be perceived and understood from the inside (Blanke, 2012). Having such understanding of the unique status of the body as an object, the study of the way individuals embody objects has been carried out using experimental paradigms involving fake limbs, like the rubber hand illusion, and immersive environments in virtual reality.

### The Rubber Hand Illusion

The rubber hand illusion is a well-established paradigm to study the sense of ownership in healthy individuals (Botvinick and Cohen, 1998; Ehrsson et al., 2004, 2005, 2008; Tsakiris and Haggard, 2005; Tsakiris et al., 2006; Marasco et al., 2011; D’Alonzo and Cipriani, 2012; D’Alonzo et al., 2014; Collins et al., 2016). In the original rubber hand paradigm, participants sit with their left arm resting on a table, hidden behind a screen, and are asked to fixate on an anatomically congruent rubber hand (Botvinick and Cohen, 1998). The experimenter simultaneously stroked the participant’s hand and the fake hand with two paintbrushes and then quantified the effect on affective embodiment by having participants respond to a questionnaire that included nine perceptual effects on a seven-point Likert scale ranging from ‘agree strongly’ to ‘disagree strongly.’ Statements included items such as “It felt as if the rubber hand were my hand” and “It seemed as if I were feeling the touch of the paintbrush in the location where I saw the rubber hand touched” (Botvinick and Cohen, 1998). After 10–15 s of synchronous visuo-tactile stimulation (less than 300 ms of temporal stroking discrepancy), participants reported significantly stronger agreement with such statements as compared to their self-reports after asynchronous stimulation, confirming a vivid sense of ownership over the rubber hand (Ehrsson et al., 2004, 2005; Lloyd, 2007; Shimada et al., 2009).

In addition to eliciting agreement with ownership statements like those above, there were also changes in the feeling of hand position (proprioception), which occurred between 10 and 60 s (Ehrsson et al., 2004, 2005; Lloyd, 2007). When asked to point with their hand where their real hand’s location is, participants tended to locate it as being closer to the non-bodily object or rubber hand, that is, participants exhibited the *proprioceptive drift* (Botvinick and Cohen, 1998; Ehrsson et al., 2004, 2005; Longo et al., 2009; Kalckert and Ehrsson, 2014). The idea is “proprioception drifts rapidly in the absence of vision, and in the [rubber hand illusion] set-up this results in overwriting the proprioceptive location information of one’s own hand with the visual location information of the rubber hand” (Kammers et al., 2009b, p. 205).

Proprioceptive drift is supported by affective measurements on the real and fake hand. One study found a decrease in the real hand’s skin temperature and a touch dulling effect (Moseley et al., 2008), although the result was not replicated in other studies (Rohde et al., 2013; De Haan et al., 2017). Also, the effects of somatosensory inputs to the real hand are reduced, leading to a lower intensity sensation (Folegatti et al., 2009; Zopf et al., 2011b; Kilteni and Ehrsson, 2017). Further, TMS elicited motor evoked potentials are reduced, suggesting the motor system is less activated toward the real hand’s muscles (Della Gatta et al., 2016). Threatening the fake hand also elicits strong cortical startle responses (Ehrsson et al., 2007; Gentile et al., 2013).

Some studies show that proprioceptive drift can also be experienced while the participant’s real hand maintains normal kinematics, that is, although the conscious sense of hand location is affected, reaching movements are not (Kammers et al., 2009a). According to these studies, even with perceptual or body image changes (i.e., a rubber hand instead of the real hand) the body schema seems to be left unaffected by the drift. This may show that there are different mechanisms for moving the body and judging bodily properties or a dissociation between the body image and the body schema. Also, it might suggest that vision of a body part does not override the elements of proprioception that maintain kinematic functions. Further, this shows a major difference between perceptual embodiment, which consists in object representation within the body image, and motor embodiment, which consists in object representation within the body schema (de Vignemont and Farnè, 2010). While the location of the rubber hand is perceptually embodied, i.e., it is similarly processed as a body part for perceptual tasks, it is not motorically embodied. However, some rubber hand experiments have found decreased motor performance with the real hand (Heed et al., 2011), which seems to entail that the motor system and perceptual judgments are both affected and leaves the question regarding the relationship between body image and body schema still open.

An interesting aspect of the study of proprioception with the experimental setting of the rubber hand illusion is the possibility of using neurophysiological evidence to enrich behavioral evidence and the subjective reports in order to address open questions. For instance, some studies have used evoked potentials (Zeller et al., 2011, 2015) or functional imaging (Limanowski and Blankenburg, 2015) to find out whether the mentioned conflict between vision and proprioception can be resolved. For example, using EEG, researchers have found an amplitude reduction of early evoked potential components over the contralateral somatosensory cortex during the rubber hand experiment (Zeller et al., 2015) suggesting a reduced connectivity in the that cortex (Zeller et al., 2016). Also, not only do schizophrenic patients have a stronger and faster (five times than normal control subjects) onset of the rubber hand experiment, but they also have long latency evoked somatosensory evoked responses (Peled et al., 2000). In a different example, experimenters have explored the role of the premotor cortex and intraparietal sulcus in the sense of ownership also using electroencephalography (Rao and Kayser, 2017). Further, the neural mechanisms, i.e., the multisensory areas of the premotor and intraparietal areas, underlying the experiment have been observed by fMRI in both healthy participants and upper limb amputees (Ehrsson et al., 2004, 2005; Bekrater-Bodmann et al., 2012; Brozzoli et al., 2012; Schmalzl et al., 2013).

Given these results, the rubber hand illusion illustrates that a non-bodily object can be incorporated into both the body image and schema. The brain appears to have modified the body image, possibly causing the proprioceptive drift, so that the participant experiences limb ownership over the rubber hand (Tsakiris and Haggard, 2005). This directly affects the body (motor) schema in that it now displays the real hand as having acquired the position of the fake one (Botvinick and Cohen, 1998; Ehrsson et al., 2004, 2005). Due to the need for a stable body image, the real hand becomes disembodied and the non-bodily object becomes part of the body image. This results in the body (somatosensory) schema being modified since touch is often reported to be felt in the rubber hand (Botvinick and Cohen, 1998; Durgin et al., 2007). The model proposed by Botvinick and Cohen (1998) understand this phenomenon in terms of the malleability of the body representation caused by multisensory processing.

The extent to which non-bodily objects are incorporated into the body image and the body schema can be further measured (Armel and Ramachandran, 2003). For instance, in one study researchers placed a Band-Aid on the participants’ real hand and a table. Researchers then stroked participant’s hidden, real hand while stroking the table at the same time (Armel and Ramachandran, 2003). To quantify the effect of affective embodiment, researchers used questionnaires and skin conductance responses, an objective measure of changes to skin electrical conductance that occur from the anticipation of pain (Armel and Ramachandran, 2003).^3^ The researchers found that participants had strong skin conductance responses when the table was threatened, i.e., the table’s Band-Aid was partly pulled off, verifying the participants’ questionnaire responses that they felt the table was their hand (Armel and Ramachandran, 2003). Also, participants “frequently reported that the table illusion was vivid when the touch was received through a common covering—the band-aids- but weak in its absence (Armel and Ramachandran, 2003, p. 1505). This suggests that consistency in the seen and felt touch is critical for body ownership. Neuropsychological evidence also confirms that the body image and body schema can be affected by inanimate objects. In one case, a brain-lesioned patient did not experience a sense of ownership toward their arm, hand, and wedding ring on the hand. However, when the ring was removed, the patient viewed it as their own (Aglioti et al., 1996). Such findings indicate the body image can be affected by higher cognitive processes.

In slight opposition to Botvinick and Cohen’s model (1998) and Armel and Ramachandran (2003) proposed the previous results were due to Bayesian perceptual learning based on the strong likelihood that stroking the real hand has caused the multisensory stimulus and not two different events, i.e., the synchronous stroking of the real hand and a table. The brain’s statistical correlations use vision over touch (Armel and Ramachandran, 2003) resulting in the viewed table becoming part of the body image. However, this proposal has been criticized due to a focus on the likelihood of sensory data, and a failure to explain why synchronous stimulation does not *always* lead to a sense of ownership (Tsakiris and Haggard, 2005; Tsakiris et al., 2009; Tsakiris, 2010; Zopf et al., 2010). For instance, studies have found that when a hidden real hand and visible checkerboard floor of the box were brushed synchronously, the subjective reports indicated a weak or non-existent illusion (Zopf et al., 2010). Notably, the self-reports and the skin conductance responses were much lower when a table was used, suggesting a role for top–down mechanisms in the sense of ownership (Armel and Ramachandran, 2003).

Similarly, the sense of ownership was inhibited when using a wooden block and fingers (Tsakiris et al., 2009) and when participants saw a smaller rubber hand (Pavani and Zampini, 2007). The illusion is also disrupted if the hand is seen as wooden sticks (Tsakiris and Haggard, 2005) or wooden slabs (Guterstam et al., 2013), and if they see their full body as a cuboid with no limbs (Lenggenhager et al., 2007). Additionally, proprioceptive drift might not be explained by statistical correlations since the rubber hand’s position has also been found to drift toward the real hand to a location between both hands (visual drift) (Erro et al., 2018).

A different model to explain the rubber hand experiment’s results is the neurocognitive model, which emphasizes a role for multisensory processing and higher cognitive functions (Tsakiris, 2010). Under this model, the rubber hand illusion results from bottom–up processing of multisensory inputs and top–down processing or stored representations of one’s hands. The sense of ownership is due to three critical comparisons. First, the object’s visual form and the pre-existing reference body model must match (Tsakiris and Haggard, 2005; Costantini and Haggard, 2007). The “more the viewed object matches the structural appearance of the body part’s *form*, the stronger the experience of the body-ownership will be” (Tsakiris, 2010, p. 13). This is supported by numerous studies (Ehrsson et al., 2005; Tsakiris and Haggard, 2005; Haans et al., 2008; Tsakiris et al., 2009). The sense of ownership is also not affected by skin color and texture, suggesting the body model is different than the body image. In addition, the current body schema state and the object’s anatomical, structural and postural features must match (Tsakiris, 2010). The sense of ownership is elicited if the object’s posture and body part match but reduces with postural and anatomical discrepancies (Ehrsson et al., 2004; Tsakiris and Haggard, 2005; Costantini and Haggard, 2007). For instance, placing the rubber hand 180 and 90 degrees to the real hand reduces the sense of ownership (Ehrsson et al., 2004; Tsakiris and Haggard, 2005; Kalckert and Ehrsson, 2014). Furthermore, the seen and felt touches of the brush must match for the sense of ownership to be elicited (Tsakiris, 2010). Notably, a recently discovered constraint is that exteroceptive signals must be integrated with interoceptive signals, such as from the cardiovascular system (Tsakiris, 2017). These comparisons suggest that for objects to be embodied into the body image, they need to be compatible with our unconscious body schema, such as postural congruency, but not with our current body image.

And yet, Guterstam et al. (2013) observed that participants embodied a volume of empty space, while others found they embodied a rubber hand 3 cm larger than their real hand (Pavani and Zampini, 2007) and three times as long as their real arm (Kilteni et al., 2012). Also, in one study synchronous visuo-tactile stimulation of one real and two rubber hands led participants to feel touch on and ownership toward, although less vividly, two right-handed rubber hands (Ehrsson, 2009). Other studies have found the sense of ownership toward two left hands (Newport et al., 2009) and four hands (Chen et al., 2018).

Rubber hand experiments have also elicited a sense of ownership with visuo-thermal stimulus patterns (Trojan et al., 2018), without vision (Ehrsson et al., 2005; Petkova et al., 2012), and without touch by using a laser pointer (Durgin et al., 2007). Thus, the phenomena are clearly more complex than can be accounted for by simple visuo-tactile integration, and researchers have accordingly moved beyond simple tactile stimulation.

Studies have also provided participants the ability to move the fake limb (Tsakiris et al., 2006; Dummer et al., 2009; Sanchez-Vives et al., 2010) to generate affective embodiment, while others have investigated motor embodiment through questionnaires (Kalckert and Ehrsson, 2012, 2014; Jenkinson and Preston, 2015). In one study, participants could move the rubber hand’s index finger (Kalckert and Ehrsson, 2012). Researchers manipulated the synchrony between the real and fake hand’s finger movements, the mode of movement (passive vs. active), and the rubber hand’s positioning. They found that both affective and motor embodiment are distinct since asynchrony eliminated both, but only motor embodiment was abolished with passive movements, while affective embodiment was reduced with incongruent positioning. Another study also found support for a double dissociation between motor and affective embodiment (Marotta et al., 2017).

Because of these complexities and variety of results, researchers have also begun to explore embodiment in virtual reality, which allows for a greater variety of experimental setups than the simple rubber hand device permits. It is to a review of that literature that we now turn.

### Embodying Objects in Virtual Reality

Virtual reality technology creates an interactive environment for its users by way of a diverse number of devices such a head mounted display (HMD), head tracking, real time motion capture, tactile feedback, and audio. To study object embodiment in virtual reality, researchers have used limb illusions (Slater et al., 2008), full body illusions using avatars (Slater et al., 2010), or combined virtual and non-virtual reality conditions (Ijsselsteijn et al., 2006). In one virtual rubber hand experiment that was set up similar to Botvinick and Cohen’s (1998) experiment, questionnaire responses and proprioceptive drift showed that participants had a sense of ownership toward the virtual hand (Slater et al., 2008).

In another study, spinal cord injury patients received tactile back stimulation while viewing virtual legs being touched through an HMD (Pozeg et al., 2017). Studies have found that there is cortical reorganization after spinal cord injuries and the lower back is connected with the leg representations (Wrigley et al., 2009). For this reason, the researchers manipulated the synchrony between the stroking of the virtual legs and the participant’s lower or upper back. The results showed that these patients experienced weaker leg ownership than healthy control participants, with the time since injury also negatively correlated with leg ownership. This suggests that these patients less readily integrate the available visual and tactile information to experience leg ownership. There was also no difference between the lower and upper back conditions, suggesting that instead of a reorganization of the primary somatosensory cortex (S1), that maybe other leg representations are involved or there are larger receptive fields on the back in S1 so that the upper and lower back conditions were engaging similar S1 locations.

In a different study, researchers used an unmediated condition, a virtual reality condition where a video-projection of a rubber hand and its visuo-tactile stimulation was on a flat tabletop surface, and a mixed reality condition, where the fake hand was projected but had unmediated visuo-tactile stimulation (Ijsselsteijn et al., 2006). Self-reports and proprioceptive drift showed that the non-virtual reality condition had the strongest sense of ownership. There was also a stronger sense of ownership in virtual reality than mixed reality but no difference in drift. The existence of differences between the virtual reality and non-virtual reality conditions contradicts a Bayesian learning explanation (Ijsselsteijn et al., 2006). Instead, these differences seem to support the neurocognitive model in that there is a role for top–down mechanisms that specify requirements for an object, since the physical objects would be experienced as more real. Improvements in VR technology may eventually erase this effect.

Virtual reality experiments on object embodiment typically use objective measures, such as defensive motor responses to a threat to a limb (Kilteni et al., 2012), an electroencephalogram via event-related potentials (González-Franco et al., 2014), temperature changes in the hand (Hohwy and Paton, 2010) and a cross-modal congruency task (Pavani et al., 2000; Zopf et al., 2011a), discussed in Section “Tools and Body Representations: Peri-personal Space,” below.

For example, in one study participants had to fixate on the virtual hand of the collocated avatar that had been placed on a desk (González-Franco et al., 2014). Event-related potentials were recorded when a knife attacked the virtual hand and when it struck the virtual table. The results showed that similar to non-virtual reality experiments, participants experienced a sense of ownership toward the virtual hand and body, suggesting that the body image has been manipulated to include the avatar and its virtual hand. Moreover, the strong sense of ownership found in the questionnaires correlated with larger P450 amplitudes. Also, when the virtual hand was threatened the motor cortex had Mu rhythm event-related desynchronization and there was more readiness potential (C3–C4) negativity.

Virtual reality paradigms are also used to study object embodiment for non-bodily objects. In one study, researchers synchronously tapped the participant’s forearm and collocated virtual forearm (Hohwy and Paton, 2010). After 30 s, the virtual forearm was replaced by a virtual cardboard box that was then synchronously tapped with the real forearm. The results showed that some participants believed they felt touch on the virtual cardboard box. However, the cardboard box illusion was not elicited without the preceding virtual hand induction, which may explain Armel and Ramachandran’s (2003) results since participants were exposed to the rubber hand illusion prior to the table touch (Hohwy and Paton, 2010).

Virtual reality paradigms can further expand on how flexible the body image and schema are and what constraints there are to embody an object. Virtual reality can easily stretch body parts past their normal size, such as a finger to double its size (Newport and Preston, 2010) or have one collocated limb that continuously grows in length and moves with the real limb (Kilteni et al., 2012). Virtual and non-virtual reality studies on object embodiment suggest that “the topographic representation in the primary somatosensory cortex (S1) reflects the perceived rather than the physical aspects of peripheral stimulation” (Schaefer et al., 2007, p. 700). In one non-virtual reality study, vision and somatic sensations were manipulated to elicit an illusion of an elongated arm (Schaefer et al., 2007). An artificial hand and arm were attached over the subject’s real one, extending it by about 20 cm. They found a sense of ownership over the lengthened limb by way of a questionnaire and neuromagnetic source imaging, which “revealed a corresponding modulation of S1 to the extent of feeling the arm elongated: the more the subjects felt the arm elongated, the more the cortical distance between D1 and D5 [digit 1 and digit 5 on the left hand] decreased” (Schaefer et al., 2007, p. 703). It was speculated that due to the perception of a longer arm, there was a larger cortical arm representation and hence participants felt a longer arm. In virtual reality, however, there does not seem to be any investigations into the amount of cortical reorganization for different virtual limb lengths (Kilteni et al., 2012). Nonetheless, there seems to be a correlation between perceiving one’s body size and shape and modulations in the cortex (Schaefer et al., 2007; Normand et al., 2011; Kilteni et al., 2012; Banakou et al., 2013; Won et al., 2015).

If there is a top–down perceptual body model and virtual reality experiments give us some insight into the embodiment of objects, then it seems the specific bodily features that it encodes are unclear. Researchers have found that the participants can embody a virtual arm that is triple in length (Kilteni et al., 2012), an avatar of a different race (Peck et al., 2013), and an avatar with a different shaped body (Normand et al., 2011; Won et al., 2015). Adult participants have also embodied a child body (Banakou et al., 2013), and feel identified with realistic androids (Cooney et al., 2012) and unrealistic humanoid robots (Aymerich-Franch et al., 2015). Such virtual reality studies appear to contradict other studies that had used a wooden stick (Tsakiris and Haggard, 2005), a rubber sheet (Haans et al., 2008), and a wooden slab (Guterstam et al., 2013) and found a reduced or eliminated sense of ownership. While a smaller sized hand was shown to inhibit the embodiment (Pavani and Zampini, 2007), other studies showed the embodiment of 30 to 400 cm virtual dolls (Van der Hoort et al., 2011) and full-body mannequins (Petkova and Ehrsson, 2008; Petkova et al., 2011).

To explain these results, functionality may be the key feature of the body model (Aymerich-Franch and Ganesh, 2016). The brain perceives an object as one’s body part or whole-body if the object’s physical properties are sufficient to allow certain actions (Aymerich-Franch and Ganesh, 2016). For instance, the rubber hand and full body illusions are possible even when rubber hands are of different color (Longo et al., 2009) or texture (Haans et al., 2008), and when the body is a different in size, gender, or age since these features do not affect proper functions (Petkova and Ehrsson, 2008; Van der Hoort et al., 2011; Maister et al., 2015). Multiple rubber hands (Ehrsson, 2009), longer arms (Kilteni et al., 2012) and larger rubber hands (Pavani and Zampini, 2007) maintain functionality, while a wooden stick (Tsakiris and Haggard, 2005), a wooden slab (Guterstam et al., 2013), and smaller rubber hands (Pavani and Zampini, 2007) cannot adequately perform tasks.

The variety of embodiment effects, as summarized by this review, seems to suggest that there is not one physical body representation. Instead, it could be the case that these observed embodiment effects are due to the interaction of multiple body representations. Indeed, there is no consensus yet on how many different bodily representations there are, what their characteristics are, and whether and to what degree they can be truly dissociated.

## Embodying Tools

Objects are those things that are nameable, identifiable, stable, and can persist through time, such as pencils and cars. Tools are a specific kind of object employed to alter or interact with other objects. Three categories of tools are physical-interaction tools, which interact with the environment; pointing tools, such as a laser; and detached tools, such as a computer mouse (tool) that interacts with objects via an interface (Holmes and Spence, 2005). Tools are different from other objects since they are associated with specific manipulation-actions, such as grasping a hammer and manipulating a nail, used to reach for objects, and used to sense one’s surroundings. This suggests a major role for the sensorimotor system in tool embodiment. They are also different from other objects since individuals typically lack a sense of ownership toward a tool. For Botvinick (2004), “the feeling of ownership that we have for our bodies does not extend to, for example, the fork we use at dinner” (p. 783). As a result, tool embodiment studies do not typically use affective measures, such as using a threat to measure physiological responses, since tool use can involve dangerous situations, such as stirring hot water.

Tool embodiment research focuses on the spatial modulation of multisensory integration following tool use, which involves *spatial embodiment*, and the plasticity of body representation following tool use, which involves *motor embodiment* The incorporation of a tool into the body and the expansion of peri-personal space are similar in that they seem to be due changes in the body schema (Iriki et al., 1996; Maravita and Iriki, 2004). But they are also separate in that the body schema requires somatic sensation, while the multisensory peri-personal space is based on vision and audition (Cardinali et al., 2009b).

### Tools and Body Representations: Peri-Personal Space

Peri-personal space is located directly around the body and involves multisensory integration in the frontal and parietal lobes (Làdavas and Serino, 2008; Serino, 2019). Evidence suggests that peri-personal space representations are multimodal since they respond to visual information (Longo and Lourenco, 2006) and visuo-tactile information (Noel et al., 2015). Also, bodily dimensions such as body part size changes the size of peri-personal space. For instance, individuals with longer arms tend to have more peri-personal space (Longo and Lourenco, 2007).

Peri-personal space has been divided into near and far space or the space within and beyond reach (Berti and Frassinetti, 2000; Witt et al., 2005). Here, reachability may be a perceptual metric such that everything that is reachable is perceived to be in action space (Witt et al., 2005). Tool use also seems to alter the perception of the environment bringing objects as perceived as farther away as closer and into the action space (Witt et al., 2005), although others have found that near peri-personal space appears to grade off from the body in that it does not abruptly end at arm’s reach (Longo and Lourenco, 2006, 2007). Studies with monkeys have shown that near space and far space are coded differently in their brain (Colby et al., 1996). Also, one PET study showed that during tool use the monkey’s brain showed an increase in the activity and reorganization of the reach representation in the intraparietal region, basal ganglia, premotor cortex, and cerebellum (Obayashi et al., 2001). These results are further supported by human behavioral studies (Berti and Frassinetti, 2000; Maravita et al., 2003; Farnè et al., 2007), PET studies (Weiss et al., 2000) and TMS studies (Lane et al., 2011). Similarly, a PET study on humans showed that during tool use the ipsilaterial posterior parietal cortex was activated (Inoue et al., 2001).

To understand what happens to the peri-personal space during and after tool use with a non-threatening object, researchers trained macaque monkeys to control a rake via a computer screen to reach food placed in far space (Iriki et al., 1996). Reachability was used when macaque monkeys were taught to reach with a rake, which extended their reach (Iriki et al., 1996). This suggests that some visual neurons code reach and adapt to changes in reachability due to tool use. They found that by recording premotor or posterior parietal cortices, tool use expanded the receptive fields of bimodal neurons to include the tool’s entire length. The enlargement of peri-personal space has been interpreted as a remapping of farther away objects as nearer ones (Di Pellegrino et al., 1997; Farnè and Làdavas, 2001). Additionally, when the macaque monkeys passively held the rake, the peri-personal space shrank back to pre-tool size. These results suggest that deliberative action is needed to expand peri-personal space (Iriki et al., 1996; Farnè et al., 2005).

In the neuropsychological literature, peri-personal space is understood as the changes in patients’ perceptual performance as stimuli are moved further away. These changes are grounded in the activity of multisensory neurons, which “decreases as the distance between visual stimuli and tactile stimuli increases” (de Vignemont, 2011, p. 85). Brain injured patients typically experience extinction, which results in contralesional events being “perceived in isolation, yet are missed when presented concurrently with competing events on the ipsilesional side” (Kennett et al., 2010, p. 15). Researchers have used cross-modal extinction, where extinction arises cross-modally, to study these patients’ peri-personal space. Results have shown that the location of visual stimuli, such as when it is close to a body part, can interfere with patients’ tactile performance (Farnè and Làdavas, 2000; Maravita et al., 2001, 2002; Farnè et al., 2005; Bonifazi et al., 2007). When these patients see a visual stimulus by the ipsilesional hand, it can extinguish a touch delivered at the same time on the contralesional hand (Di Pellegrino et al., 1997; Costantini et al., 2007). Typically, as the visual stimulus moves away from the hand or vice versa, tactile detection improves on the other hand (Làdavas, 2002).

Researchers have studied the effects of patients with tactile extinction using a tool on the peri-personal space (Farnè and Làdavas, 2000). They found that short periods of rake use with the ipsilesional hand to reach for objects (red fish) in far space increases extinction of tactile contralesional stimuli (hand touch) after visual stimuli (light flash) were placed close to the tool tip. This suggests peri-personal space expanded to include the rake’s entire axis and that the rake is now part of the body. Also, similar to Iriki et al. (1996), the peri-personal space returned to pretool use size after 5–10 min of passively holding the rake. Further, free hand pointing movements toward the fish had results comparable to the pretool condition. This suggests that mere motor activity is not sufficient to expand peri-hand space (Farnè and Làdavas, 2000).

In a follow-up study, Farnè et al. (2005) used the same task to research whether the absolute of functional tool length was important to modulate the peri-personal space. They used a long (60 cm) wooden rake, a short (30 cm) wooden rake, and a hybrid long (60 cm) wooden rake where the functional part was attached at 30 cm (Farnè et al., 2005). They found that after using the hybrid (60 cm) tool, the crossmodal extinction was compatible with the 30 cm short tool. This suggests that the tool’s functional part is directly related to the expansion of peri-personal space. Indeed, “the main advantage provided by the extension of the peri-hand area, whereby vision and touch are integrated, seems to be that of allocating multisensory processing where the goal of the action is” (Làdavas and Serino, 2008, pp 1106–1107). Similarly, Tomasino et al. (2012) found an increase in fMRI activity in the extrastriata body area when a joystick was used in a more compatible environment in near space than when a less appropriate tool (extended pliers) was used in a less congruent environment in near space. This finding suggests that the body’s neural representation is adapted in a functional manner, depending on tool compatibility.

Another method to assess peri-personal space, the cross-modal congruency task, tests “how strongly visual stimuli affect the processing of simultaneously presented tactile stimuli” (Holmes, 2012, p. 273). Similar to cross-modal extinction, the effect is larger when spatially incongruent distractors are near the tactually simulated hand and reduces when presented close to the opposite hand. For example, in one study participants judged whether tactile vibration was delivered to the thumb or index finger on either hand while holding two golf clubs. Two visual distractor lights were at each far end of both golf clubs (Maravita et al., 2002). Each trial consisted in a vibration from one of four possible locations (finger or thumb on either hand) accompanied with one distractor light. In the uncrossed condition, visual distractors interfered with the tactile discrimination task on the same side of the tool. When tools were crossed the visual distractors had stronger interference on the hand placed in the opposite space. Like in other studies, passively holding the tools did not have this effect.

The studies reviewed suggest that tool-use shows characteristics of tool embodiment, such that peri-personal space extension is dependent on active (Farnè and Làdavas, 2000), goal-directed (Farnè and Làdavas, 2000), and functionally effective (Farnè et al., 2005) tool interaction. Other studies also support that during and after tool use, the peri-personal space is enlarged. For example, researchers have found that using a computer mouse enlarges peri-personal hand space, which may also include the screen monitor (Bassolino et al., 2010). In another study, peri-personal space was expanded when blind individuals merely held the cane without using it Serino et al. (2007). This suggests that “blind people, who continuously use the cane to integrate auditory and tactile information in far space, in order to compensate for the lack of visual information, developed a new, extended representation of auditory peri-hand space, which is selectively activated when holding the cane” (Serino et al., 2007, p. 1108). Long term regular use with a tool then may create a durable extension of peri-personal space. Further, other studies have found that tool use imagery triggers similar effects on the peri-personal space (Baccarini et al., 2014). Notably, the peri-personal space can also contract when weights impair arm movement (Lourenco and Longo, 2009) and after limb loss (Canzoneri et al., 2013).

However, an alternative explanation for some of these results is that “tools act as spatial attentional and motor cues” (Holmes et al., 2007, p. 466). To investigate this, Holmes et al. (2004) modified the cross-modal congruency task by placing the visual distractors near the hand, the tools’ middle, and far from the hand or the tool’s tip. Results showed that the effects of tool use were mostly at the tool’s tips and weakly at the tool’s middle. They also conducted a fMRI study that showed that there was a shift in participants’ spatial attention to the functional part of the tool. They asked participants to ignore visual distractors but pay attention and respond to vibrotactile targets that were at the tool’s tip and felt in the hand. The results were indicative of a shift in spatial attention since visual distractors at the tool’s tip enhanced the BOLD response in retinotopic portions of the occipital visual cortex and decreased the BOLD response in the ipsilateral visual field (Holmes et al., 2008).

Another important research area explores the idea that tool use can make changes to the body schema. Some researchers have indicated that the peri-personal space expansion occurs because the tool is incorporated into body schema, i.e., into the sensory-motor map of the limb with the tool (Iriki et al., 1996; Farnè and Làdavas, 2000; Maravita and Iriki, 2004; Serino et al., 2007). Additionally, some studies have also shown that the tactile signals felt in the hand that occur when a held tool comes into contact with an object are referred directly to the tip of the tool (Yamamoto and Kitazawa, 2001; Maravita et al., 2002; Yamamoto et al., 2005; Collins et al., 2008). This result suggests that somatosensory integration is not required to use a tool. Hammering a nail with a hammer tends to result in the feeling of touch on the part of the hammer touching the nail, and not at the hand holding the tool. This result seems to suggest that the body (somatosensory) schema has been changed and now includes the tool’s functional part. Tool use then not only seems to have effects on peri-personal space coding but also seems to change other aspects of body representations, a topic to which we now turn.

### Tool and Body Representations: Body Schema

The peri-personal space and body schema are separate entities that are highly flexible and seem to be connected to the motor system.^4^ Some studies have found that the modulation of the body schema during tool use involves increasing the length of the arm’s representation and requires goals and motor programs (Cardinali et al., 2009b, 2012; Sposito et al., 2012; Baccarini et al., 2014; Garbarini et al., 2015). For example, Cardinali et al. (2011) found that tool use involving a 40 cm long mechanical grabber resulted in a free hand kinematic pattern compatible with having a longer arm, while there were no changes in the hand-related or grip component. This suggests that for a time after tool use the body schema was still modified as if participants were still using the mechanical grabber and had a longer arm.

In the same study, Cardinali et al. (2011) investigated whether tool use can elicit a functional update of the body schema without affecting the body image by way of a motor and perceptual task (Cardinali et al., 2011). For the motor task, blindfolded participants were asked to point with their left index fingertip to a specific location on their right arm that had been touched by the experimenter, i.e., the finger, wrist, or elbow. For the perceptual task, participants verbally reported where the experimenter had touched them. They found that after tool use participants perceived a longer arm since they localized touches to their elbow and middle fingertip as farther apart. In contrast, there was no observed change when localizing named body parts, suggesting tool use may only affect the body schema.

Other studies have used a direct behavioral task, e.g., an arm bisection task, to investigate whether tool use might alter the metric representation of limbs (Sposito et al., 2012). Researchers asked healthy participants to estimate the subjective midpoint of their own forearm before and after a training phase with long functionally relevant (60 cm) tools and small functionally irrelevant (20 cm) tools. They found that participants indicated a more distal midpoint, thus exhibiting an increased representation of the arm’s length after training with only the long functionally relevant tool.

Similarly, other studies used this task on brain-damaged hemiplegic patients with pathological embodiment (Garbarini et al., 2015). The participants were asked to estimate the midpoint of their paralyzed forearm before and after a training phase in which an experimenter repeatedly used a tool and were aligned or misaligned relative to patients’ shoulders. In the aligned condition, patients thought that they were using the tool with their paralyzed arm. In effect, there was significant modulation of the perceived arm length in that they located their forearm midpoint closer to the hand, indicating an increased length, in the post- training phase. No effect occurred when they were misaligned to the experimenter during the training phase (Garbarini et al., 2015). These “findings show the existence of a tight link between spatial, motor and bodily representations and provide strong evidence that a pathological sense of body ownership can extend to intentional motor processes and modulate the sensory map of action-related body parts” (Garbarini et al., 2015, p. 402).

Cardinali et al. (2016) also found that using tools (sticks and pliers) that elongate the fingers results in an update of the brain’s hand representation due to the tools’ morpho-functional characteristics and its specific sensorimotor constraints. They found that the kinematics of the grasping hand component were affected, but the arm representation and reaching kinematics were not (Cardinali et al., 2016). Similarly, Miller et al. (2014), used a perceptual task to find that the hand representation increased in size when using a tool morphologically similar to the hand. They also found that the use of an arm-like tool affects its representation by an increase in arm length (Miller et al., 2014). Therefore, the plasticity of the limb’s representation is constrained by the resemblance of the tool’s morphology.

Studies have also found that the peri-personal space and the body schema can be separated. One study found that after immobilizing participants’ right limb for 10 h, that the limb had a reduction of peri-personal space but no effect on the body schema since there were no consequences on the perceived limb’s length. They also found the left overused limb’s peri-personal space did not extend while the perceived length increased (Bassolino et al., 2015). These results suggest that both the peri-personal space and body schema depend “on different mechanisms; while [peri-personal space] representation is shaped as a function of the dimension of the acting space, metric characteristics of [body representation] are forged on a complex interplay between visual and sensorimotor information related to the body” (Bassolino et al., 2015, p. 385).

Further, these tool embodiment studies show a role for both somatic sensations and vision in the incorporation of a tool into the body schema. Somatic sensations seem to be necessary and sufficient for tool incorporation into the body schema. In one study using blindfolded participants, Martel et al. (2019) concluded that participants assessed arm length representation at an implicit level by comparing movement kinematics before and after tool use. The results showed that after tool use participants had modified movement kinematics that suggested an increased arm length representation. Martel et al. (2019) also found that “explicit arm representation seems immune to tool-use when only somatosensation is available. When participants were asked to explicitly estimate their arm length, we observed no effect of tool use” (p. 10). Neuropsychological evidence also supports this in the case where a deafferented patient had no incorporation of a tool due to a lack of proprioception (Cardinali et al., 2016).

### Tools and Virtual Reality

As in the case of embodying objects, virtual reality provides a good medium to test different measures and to investigate how interacting with virtual tools might compare to their physical counterparts. Studying the expansion of peri-personal space and body schema changes in virtual reality can provide important insights into how these environments effect our movement planning and execution.

The cross-modal congruency task has also been used in virtual reality to test whether peri-personal space can change with virtual robotic tools (Sengül et al., 2012, 2013). Like in Maravita et al.’s (2002) experiment with physical golf clubs, the task was to cross or uncross the virtual golf clubs (in one experiment) and to just hold the tool interface (in the other experiment), where the experimenter did the crossing and uncrossing (Sengül et al., 2012). The latter experiment aimed to explore whether active use of the tool was important to spatial modulation of the crossmodal congruency effect. Subsequently, participants made discriminations of vibrotactile stimuli delivered to the thumb and index finger using a foot pedal while ignoring visual distractors at the virtual tool’s tip. Results showed that virtual-robotic tools can influence multisensory integration in peri-personal space. Moreover, “there was an interaction of vision and touch as reflected in the cross-modal congruency effect (CCE) for virtual robotic tools. Second, it was found that actively crossing the tool resulted in a remapping of peri-personal space, as reflected in a stronger CCE when visual stimuli appeared at a different side than the tactile vibration, at the tip of the tool that was held in the stimulated hand. Third, it was found that this remapping of peripersonal space did not depend on active tool use, as passive crossing of the tools resulted in a change in the CCE side effect” (Sengül et al., 2012, e49473). Like in other non-virtual reality studies then, active crossing of tools results in remapping the peri-personal space at the tool’s tip. However, unlike some non-virtual reality studies, they found that active tool use was not required for the remapping of peri-personal space.

In the context of virtual reality, another study demonstrated that body metric estimation can be modulated by *motor embodiment* (D’Angelo et al., 2018). Participants were asked to perform a forearm bisection task before and after a training phase, in which they virtually grasped objects on a PC screen with a virtual hand. Researchers used a leap motion controller, a virtual reality device used for hand tracking, to synchronize the virtual hand to the participant’s real hand. In a synchronous condition, participants had collocated virtual hand movements to their own right-hand movements, while in an asynchronous condition had a 3 s delay between the participants’ real hand and the virtual hand movements.

The results seem to confirm that the plastic changes of peri-personal space and the body schema “depend on the experience of controlling the course of events in space through one’s own actions, i.e., the sense of agency” (D’Angelo et al., 2018, p. 1). Participants pointed to their forearm midpoint more distally, consistent with an increased length of the arm representation, after performing the training phase in the synchronous condition only. This suggests that having a different body metric depends on the intention to perform the action being congruent with the movement’s motor output. These findings suggest “that body schema and peri-personal space are affected by the dynamic mapping between intentional body movements and expected consequences in space” (D’Angelo et al., 2018, p. 1).

These studies may be taken as preliminary evidence for virtual tool embodiment. Similar to object embodiment, virtual reality paradigms have the potential to further expand on how flexible the body schema is and what constraints there are to embody a tool. Virtual reality can easily stretch a tool past its original size to see the effects on peri-personal space and the body schema, while also discovering the tool’s breaking point of no longer being a functioning tool for a task and the associated brain changes.

## Radically Embodied Tools and Objects

So far, we have reviewed different ways in which the embodiment of tools and objects is understood as affecting body representations. Both in the case of objects and in the case of tools, embodiment occurs when they are incorporated into either the body image (consciously) or into the body schema (somewhat unconsciously). Moreover, we have seen research based on paradigms such as the rubber hand illusion and virtual reality to assess embodiment of different elements and of in different forms. In this section, we will sketch the issue from the point of view of radical embodiment. As a caveat, it is important to note that the embodiment of objects and tools from the point of view of radical embodiment is not essentially different from the one already reviewed. At the end of the day, the radical embodiment of objects and tools also consists of taking extra-somatic parts of our environment to be proper parts of our body. The differences with the framework based on body representations come from the very characterization of such an embodiment in non-representational terms and from some radical consequences that follow from this move. We will turn to these topics but, first, we succinctly introduce radical embodiment.

### A Primer on Radical Embodiment

We use the umbrella term of *radical embodiment* to refer to a group of theories of perception, action, and cognition inspired in phenomenology (Gallagher and Zahavi, 2007; Käufer and Chemero, 2015), ecological psychology (Gibson, 1966, 1979; Michaels and Carello, 1981; Richardson et al., 2008; Chemero, 2009; Turvey, 2019), and enactivism (Varela et al., 1991; Hutto and Myin, 2013; Di Paolo et al., 2017), as well as to some radical proposals that follow from the hypothesis of the extended mind (Menary, 2010; Kirchhoff and Kiverstein, 2019). Although we are well aware that there are more or less important differences between all these approaches (Walter, 2010a, b), we are not going to take issue with them as, for the sake of our study, their similarities overrule their differences.

The features of radical embodiment relevant for our discussion are its commitment to anti-representationalism and its characterization of cognitive systems as complex, self-organized dynamical systems. Cognitive systems are not taken to be in the business of constructing internal representations of the external environment to be able to deal with the latter. On the contrary, cognitive systems are already embodied, embedded systems which are in the business of maintaining their internal dynamics in harmony with their dynamical interaction with the environment. According to radical embodiment, such a fundamental relation between cognitive systems and their environments makes the need for internal representations of the latter either superfluous (Brooks, 1990; Chemero, 2009) or unintelligible in most of the cases (Hutto and Myin, 2013). Mental representations are supposed to play an informative role in cognition. They are the elements cognitive systems use to *make epistemic contact* with their surroundings. Once that contact is granted through embodiment and embedment and explained in terms of dynamical interactions, the need for mental representations disappear.

For the topic of this article, the consequences of radical embodiment are pretty straight forward: the embodiment of objects and tools is not a matter of incorporating them in body representations. Although nobody denies that the brain carries information about the states of our bodies and environment (and thus in this minimal way “represents”), for radical embodiment this fact is a trivial background condition for the possibility of adaptive behavior and is not in and of itself explanatory. Thus, the embodiment of objects and tools must be explained in some other terms. The question is which ones. In the following we try to sketch them.

### Tools of Radical Embodiment

The philosophical works of Martin Heidegger and Maurice Merleau-Ponty are useful to get a sense of the notion of tool and object embodiment from the coordinates of radical embodiment. In a famous passage of *Being and Time*, Heidegger (1962/2001) considers the foundational aspects of the use of tools. According to him, tools are never meaningless chunks of matter that must be incorporated into our meaningful interactions with the environment by some kind of reflective effort. Rather, we typically encounter tools within our purposive engagements with the world. In this sense, tools are typically about us and about many things in many interrelated ways.

First, a tool—or *equipment* in the Heideggerian jargon—is always something about what it is for and about the kind of practice or task in which it is used. For this reason, a tool is always about the capacities and interests of the user—the *Dasein*—and about other tools and objects that belong to the same kind of practices (see, e.g., Heidegger, 1962/2001, p. 97). A pan is for cooking, but a specific kind of cooking, one that requires oil and fire rather than, say, citrus juices, *ají*, and salt as in the case of ceviche. As such, a pan is the kind of tool it is by virtue of its role in the specific practice of cooking in which it is used; that is, a pan only make sense as a pan when it is on a fire, with oil, with some kind of food within it, etc.

As important as fire, oil, and food for the pan to be a pan are the abilities and interests of the users of pans. Heidegger claims: “The work produced [by tools] refers not only to the ‘toward-which’ of its usability and the ‘whereof’ of which it consists: under simple craft conditions it also has an assignment to the person who is to use it or wear it” (Heidegger, 1962/2001, p. 100). In other words, a tool is by itself always about the user: there is always a meaningful relationship between tools and users just by virtue of being tools and users. A tool is about users in that it is for them to do something with it that is “for-the-sake-of” and determined by the “totality of [their] involvements” (Heidegger, 1962/2001, p. 116). Such a referential aspect of the involvement of users and tools inspires a non-representational understanding of tool use and embodiment. In a famous locution, tools are *ready-to-hand* for the Dasein (Heidegger, 1962/2001, p. 91 & ff.); namely, users engage with tools and tools are incorporated into the users’ dynamics without requiring any conceptual work from them. Tools are ready to be used and, when that happens, they become tacitly integrated into the user. Such is the Heideggerian way to refer to something equivalent to tool embodiment.

The key aspect of Heidegger’s point regarding tools is that they can become embodied without the need for conscious reflection. *Prima facie*, such an approach is compatible with a strong form of anti-representationalism: no need for conscious assessment of tool embodiment means no need for a conscious mental representation into which the tool must be integrated. In this sense, Heidegger seems to avoid the need for a body image to understand tool embodiment. However, his proposal still seems to be compatible with some notion of body schema, as it is unconscious by definition (Gallagher, 2000). Moreover, the concept of body schema itself has been used within the phenomenological (and Heideggerian) tradition. For example, Merleau-Ponty (1945/2012) uses the notion of *schema corporel*, which he also calls the “lived” or “habitual” body, to refer to the relevant understanding to of the body to capture human behavior and engagement with the different elements of the environment.

Given the theoretical compatibility between the phenomenological tradition that is at the roots of radical embodiment and the notion of body schema usually entertained in the representational framework of object and tool embodiment, there must be something else to distinguish between the two frameworks. As the notion of body schema is ambiguous regarding its representational character—i.e., it can be understood in terms of representations, like in the representational framework of object and tool embodiment, and in non-representational terms, like in the case of Heidegger or Merleau-Ponty—the non-representational character of radical embodiment must be manifest in other considerations. If this were not the case, the distinction between the two frameworks would be a matter of linguistic preference if not directly trivial. So, what are these considerations that make radical embodiment actually radical and anti-representational?

The relevant considerations are methodological. The differences between the representational framework of object and tool embodiment and the framework based on radical embodiment do not (only) lie on theoretical grounds. It is true that there are some theoretical issues and incompatibilities between them, but it is also true that notions as body schema may be a point of connection if the debate were strictly held in theoretical terms. However, the differences between both frameworks become more salient when the methods to assess tool embodiment are studied. As we have seen in previous sections, when it comes to understanding the embodiment of tools in terms of body image and body schema the methods used have to do, for instance, with the illusions provoked by the rubber hand paradigm, with reaction times, with personal reports, etc. In the case of radical embodiment, the measurements of the embodiment of object and tools are completely different and have to do with the tools provided by complexity science (Riley and Van Orden, 2005; Holden et al., 2013).

As we have noted in the previous section, from the point of view of radical embodiment, cognitive systems are complex, self-organized dynamical systems. This amounts to saying that cognitive systems are systems that impose their own order (aka organization) on themselves by virtue of the ongoing interactions between their components and of the ongoing interactions of the whole system with its environment. Given this, it is commonly acknowledged that this kind of system exhibits some specific patterns of complexity by virtue of their self-organization and the foundation on multiple interactions at multiple scales such as organization around critical states and fractal features (Juarrero, 1999; Riley and Turvey, 2002; Van Orden et al., 2003; Carello and Moreno, 2005; Stephen and Dixon, 2009; Kuznetsov et al., 2013; Lamb and Chemero, 2013).

The features of complex, self-organized dynamical systems have been studied both to determine the kinds of activities cognitive systems are involved (Holden et al., 2009) and to characterize and individuate cognitive systems themselves (Van Orden et al., 2005). In the latter context, these features are relevant to understand object and tool embodiment. The underlying idea is that, when an object or a tool is embodied in a cognitive system, the tool becomes a proper part of the cognitive system and, therefore, the *whole* system (e.g., human body + tool) must exhibit the expected signatures of self-organization and complexity, such as a fractal organization of its different scales. In this sense, the studies of object and tool embodiment from the standpoint of radical embodiment do not really look for reaction times simpliciter or for participants’ reports of ownership. Those studies look for the features of complex, self-organized dynamical systems at the scale of the cognitive systems and the object/tool as a whole, unitary system. If those features are present, the object/tool may be said to be embodied in the cognitive system. If not, the object/tool is disembodied.

The work developed by Dotov and colleagues is a paradigmatic example of this kind of study (Dotov et al., 2010, 2017). In their studies, Dotov and colleagues study tool embodiment by explicitly addressing the Heideggerian notions of readiness-to-hand, roughly equivalent to embodiment, and presence-at-hand, roughly equivalent to disembodiment. In other words, when a tool is embodied in a cognitive system, it is ready-to-hand and, when a tool is not embodied in a cognitive system, it is present-at-hand. For example, when a person rides a bike and it works perfectly, the bike is ready-to-hand, meaning that the bike becomes embodied and the whole person-bike system exhibits the fractal features of complex, self-organized dynamical systems. When the bike stops working for some mechanical reason, for example, it becomes present-at-hand, meaning that the bike becomes disembodied and the whole-person bike system is not a unitary system anymore and does not exhibit the fractal features of complex, self-organized dynamical systems. The work of Dotov and colleagues shows that these transitions and their fractal signatures are common in our engagement with tools and take them to be examples of tool embodiment. In sum, the traditional view takes embodiment to indicate an alteration in one or more body representations; radical embodiment takes it to indicate the achievement of a particular kind of dynamic coupling.

What has not to our knowledge been studied, but should be, is the relationship between these kinds of signatures of synergistic coupling between bodies and tools, and the feeling of ownership and embodiment of, for instance, a prosthetic limb. Such work could inform prosthetic design, as it may be that the ease with which a prosthetic can be coupled to the brain-body-environment system is a better predictor of the likelihood of prosthetic embodiment [an important aspect of positive experiences with prosthetics (Anderson, 2018)] than are aesthetic design dimensions. Such work could be carried out in VR environments, which would give fine control over the dynamics of the prosthetic, allowing exploration of the different ways a prosthetic might ease or disrupt coupling, and the effect of such manipulations on experience.

## Conclusion

The study of object and tool embodiment is already a mature field in which different frameworks, methodologies, and experimental paradigms work in parallel, and sometimes together, to understand the ways and conditions under which we take objects and tools to be part of our bodies. In this paper, we have seen that such study is pursued both from a representational and from a radical embodied point of view. We have also seen that, despite the apparent incompatibilities between the two points of view, notions such as body schema may offer an important connection between them. In this sense, the difference between the representational and the radical embodied notions of object and tool embodiment is mostly methodological. While the representational point of view uses methods like personal reports, skin conductance measurements, etc., the radial embodied point of view uses the tools provided by complexity science to see whether there are tacit, non-conscious engagements with object or tools that can be labeled as embodied.

The theoretical compatibility between both points of view opens an interesting path of research in which semantic debates could be set aside (e.g., the debate on whether the body schema is a proper representation or not) and experimental work from both frameworks could inform each other to achieve a better understanding of the phenomenon and could be combined to improve future research. For example, in both tool and rubber hand studies, it is unclear whether the multisensory effects of tool embodiment can be ascribed to a change in the body schema, in the processing of the peri-personal space, and/or a shift in attention.

It has been shown that using a tool quickly modifies the perception of the peri-personal space, the kinematics of subsequent bodily movements, and the perceived size of the limb. However, this might suggest a faster mechanism, such as shift or projection of spatial attention toward the tool tip rather than a likely slower mechanism, such as tool embodiment (Holmes et al., 2007). The methods used in the radical embodied approach to embodying object and tools (e.g., Dotov et al., 2010) could help to further clarify this issue as they provide a neat way to better understand whether the body schema is involved or not in a given task—by discriminating between readiness and unreadiness-to-hand, for example.

Another field for future research has to do with the ability to use tools and have them become a part of one’s own body while interacting with them in the virtual space. It is difficult to know when a virtual tool has become embodied. Some researchers suggest that instead of questionnaires that we evaluate these technologies by using change in attention as a measure of how people interact with virtual environments (Dotov et al., 2010). Again, the methodological approach of radical embodiment could be used to gain better understanding of this issue.

There are also some non-virtual reality tasks that could be used in virtual reality to study the flexibility of the body representations and the somatosensory cortex, such as with the elongation of a limb (Kilteni et al., 2012), and the brain areas responsible for object and tool embodiment. Further work could be done on the amount of cortical reorganization under various lengths of the virtual limbs for object embodiment and tools for tool embodiment. More research could be on how fast or gradual the peri-personal space reduces after tool use and how fast the cortical reorganization and its reduction is. Future studies could also expand on our understanding of clinical populations and, for instance, their amputated arms or legs by way of objective measures, such as fMRI, in both non-virtual reality and virtual reality rubber hand and full body paradigms. Also, future studies are needed to better understand the use of a robotic touch interface (Marasco et al., 2011) and its effects on the sense of ownership over a prosthetic limb in both non-virtual reality and virtual reality paradigms.

Understanding the underlying brain mechanics is crucial for a full understanding of how object and tool embodiment can work with body representations. Further work on studying experienced and inexperienced tool users with participants in ecologically valid non-virtual and virtual settings will expand the tool use literature. Additionally, further methods to study the body image during tool use should be explored. Continuing to research on tool and object embodiment may allow for the development of more effective prosthetics for missing limbs. In any case, all these are open questions that can be addressed from the framework based on body representations or from a different one.

## Author Contributions

All authors listed have made a substantial, direct and intellectual contribution to the work, and approved it for publication.

## Conflict of Interest

The authors declare that the research was conducted in the absence of any commercial or financial relationships that could be construed as a potential conflict of interest.

## Footnotes

1 As we will detail below, the number and types of body representations that exist is a matter of dispute. See Anderson (2018) for discussion. For our purposes we will be content with the standard distinction between body image and schema.

2 It is worth noting that de Vignemont (2011) refers to affective, motor, and spatial embodiment as *implicit measurements* of embodiment. We do not use this wording in our article because we take them to be aspects of a conceptual analysis of embodiment that can actually be mapped onto the senses of ownership, agency, and self-location respectively. Also, we prefer to use the wording of “measurements” for the explicit measurements in the literature. We want to thank an reviewer for pointing out this fact and giving us the opportunity to clarify it.

3 Other experiments studying affective embodiment have also used skin conductance responses using a similar experimental setting (Ehrsson et al., 2007; Guterstam et al., 2011).

4 It is worth noting that, despite this classical view regarding the differences between peri-personal space and body schema, some researchers have suggested they are just two labels for the same concept (Cardinali et al., 2009a).
